# Effect of the Critical Shoulder Angle on the Efficacy of Ultrasound-Guided Steroid Injection for Subacromial Bursitis

**DOI:** 10.3390/jpm12111879

**Published:** 2022-11-09

**Authors:** Che-Li Lin, Ming-Ta Yang, Yu-Hao Lee, Hung-Chou Chen, Yi-Wen Chen, Timporn Vitoonpong, Shih-Wei Huang

**Affiliations:** 1 Department of Orthopedic Surgery, Shuang Ho Hospital, Taipei Medical University, Taipei 23561, Taiwan; 2 Department of Orthopedics, School of Medicine, College of Medicine, Taipei Medical University, Taipei 11031, Taiwan; 3 Center for General Education, Taipei Medical University, Taipei 11031, Taiwan; 4 Clinical Research Center, Taipei Medical University Hospital, Taipei 11031, Taiwan; 5 Department of Physical Medicine and Rehabilitation, Shuang Ho Hospital, Taipei Medical University, Taipei 23561, Taiwan; 6 Department of Physical Medicine and Rehabilitation, School of Medicine, College of Medicine, Taipei Medical University, Taipei 11031, Taiwan; 7 Rehabilitation Department, King Chulalongkorn Memorial Hospital, Bangkok 10330, Thailand

**Keywords:** subacromial bursitis, steroid, ultrasound, critical shoulder angle

## Abstract

The critical shoulder angle (CSA) is associated with impingement and rotator cuff lesions, and ultrasound-guided corticosteroid injection is effective for subacromial bursitis. However, because the efficacy of this treatment varies, this study investigated the effect of the CSA on the efficacy of corticosteroid injection in the subacromial space. Patients who received a diagnosis of subacromial bursitis after a clinical physical examination and ultrasound were enrolled prospectively from May 2019 to December 2021. Patients’ baseline variables and CSAs were assessed before intervention. Patients’ shoulder pain and disability index (SPADI), visual analog scale (VAS), and shoulder joint range of motion (ROM) scores were assessed at 2, 6, and 12 weeks after ultrasound-guided corticosteroid injection. All participants were divided into CSA > 38° and CSA ≤ 38° groups. We conducted the intragroup and intergroup comparisons of the variables and performed Pearson analysis to identify potential correlations between the CSA and outcome parameters. A total of 55 patients were enrolled in this study. Of these, 28 were included in the CSA > 38° group and 27 in the CSA ≤ 38° group. The baseline variables of the two groups did not differ. In the intragroup and intergroup comparisons, although VAS, SPADI, and ROM scores improved up to 12 weeks after intervention, no difference was identified between groups. The Pearson analysis revealed a positive correlation (r = 0.30, *p* = 0.024) between the CSA and VAS scores before the intervention. However, no correlation was found between the CSA and follow-up parameters. The CSA was not associated with the clinical efficacy of ultrasound-guided corticosteroid injection for subacromial bursitis.

## 1. Introduction

Subacromial bursitis is characterized by inflammation in the subacromial space caused by the repeated impingement of the subacromial-related tissue structures of the subacromial space and proximal humerus [1]. Subacromial bursitis causes pain in the anterolateral portion of the shoulder and commonly causes disability [2]. Up to 65% of patients who experience shoulder pain and visit general practitioners receive a diagnosis of subacromial bursitis and impingement [3]. The bursa is a fluid-filled sac that provides cushioning and lubricates adjacent structures to reduce stress and friction caused by joint motion. The subacromial bursa is located under the acromion and deltoid muscles and above the supraspinatus tendon. Pain in this area generally occurs due to the compression of the bursa through the abduction or internal rotation of the shoulder, and such pain can be reproduced using the empty can, Neer, or Hawkins impingement tests [2,4]. In addition to clinical diagnosis, impingement and subacromial bursitis can be diagnosed through ultrasound and magnetic resonance imaging (MRI); both have demonstrated high diagnostic accuracy [5,6].

Chronic subacromial bursitis generally causes persistent pain without spontaneous recovery. Treatment for chronic subacromial bursitis includes anti-inflammatory medication, physical therapy, and subacromial local injections. Among these treatments, subacromial corticosteroid injections are most commonly used as standard treatment because of their anti-inflammatory effects, which can lead to shoulder pain relief and function improvement [7]. For traditional subacromial injections, anatomical landmarks are used to guide the selection of an injection point. However, subacromial injection is more commonly administered under ultrasound guidance because of the increasing usage of ultrasound in clinical application, and this procedure may be more effective in patients with chronic subacromial bursitis [8]. Additionally, a systematic review and meta-analysis indicated that corticosteroid injection with ultrasound guidance was superior to palpation guidance in pain relief, range of motion, and functional improvement [9]. A Cochrane review indicated that subacromial corticosteroid injection is effective in individuals with rotator cuff disease [10]. However, another systematic review reported that findings regarding the effectiveness of subacromial corticosteroid injection in patients with subacromial impingement syndrome are inconsistent [11]. This may indicate heterogeneity with respect to the efficacy of subacromial corticosteroid injection in individuals with different clinical characteristics.

The critical shoulder angle (CSA) is a radiographic shoulder angle measurement that represents the inclination of the lateral extension of the acromion and glenoid in the anteroposterior view of shoulder X-rays [12]. The CSA is the angle formed from the line of the superior to the inferior bony margin of the glenoid and from the inferior bony margin of the glenoid to the lateral margin of the acromion. Patients with rotator cuff tears were reported to have a higher CSA than did those without [13]. In addition, studies have used the CSA to predict supraspinatus tendon tears and the risk of retears after arthroscopic repair surgery [14,15]. Another study reported that the CSA along with age can predict rotator cuff impingement and calcific tendonitis [16]. Moor et al. reported that the CSA could contribute to the risk of degenerative RCTs by affecting glenoid inclination and lateral extension of the acromion. Previous biomechanical studies have confirmed that a high CSA was associated with overloaded tensile stress on rotator cuff tendons, which then led to more vulnerable to impingement. Subacromial impingement of shoulder can lead bursitis [12,13]. On the basis of the findings of the aforementioned studies, we hypothesized that the CSA would play a key role in determining the efficacy of corticosteroid injection for subacromial bursitis, which is related to impingement syndrome. This study investigated the association of the CSA with the efficacy of ultrasound-guided corticosteroid injection in patients with subacromial bursitis. We hypothesized that corticosteroid injection would be less effective in patients with subacromial bursitis and higher CSAs.

## 2. Materials and Methods

### 2.1. Study Design

We conducted this prospective case–control cohort study in a medical university hospital. Patients with shoulder pain were recruited from the clinics of our hospital between May 2019 and December 2021. A clinical physician used ultrasound to evaluate whether eligible patients had rotator cuff problems and met inclusion and exclusion criteria. The inclusion criteria were as follows: (1) older than 20 years, (2) presence of chronic shoulder pain, (3) physical examination and ultrasound findings of subacromial bursitis with impingement syndrome, and (4) a more than 40% reduction in pain upon active shoulder abduction or internal rotation after injection of 2 mL of 1% lidocaine into the subacromial–subdeltoid bursa by ultrasound guidance. Patients were excluded if they met any of the following criteria: (1) having adhesive capsulitis as a comorbidity and a limited range of motion (ROM) of the shoulder, (2) having rupture or tear of rotator cuff, (3) having a history of undergoing joint replacement or arthroscopy surgery of the affected shoulder, (4) receiving corticosteroid, hypertonic dextrose, hyaluronic acid, or platelet-rich plasma injection or any type of injection in the shoulder joint within the preceding 3 months, (5) having a neurological disease causing weakness on the affected side and impairing cognitive function and the ability to complete the questionnaire, and (6) simultaneously participating in another clinical trial or refusing to participate in this study. The groups were divided according to participants’ CSAs. Based on our previous study, patients with a CSA of >38° have a higher risk of rotator cuff lesions (partial or full-thickness tear of supraspinatus tendon) [17]. Therefore, participants with a CSA of >38° were categorized as the high CSA group, and those with a CSA of ≤38° were categorized as the low CSA group. CSA images were obtained before ultrasound evaluation and corticosteroid injection, and the ultrasound operator was unaware of the CSA of participants. A physician who was blinded to the group of and injectant used for each participant administered corticosteroid injections into the subacromial bursa under ultrasound guidance. Moreover, the assessor of the outcome measurements was blinded to group allocation throughout the study. We obtained written informed consent from participants after explaining the study aim and procedures to them. This study was approved by the Institutional Review Board (IRB) of Taipei Medical University (IRB no. N201903058). All methods in this study were performed in accordance with relevant guidelines and regulations.

### 2.2. CSA Assessment

An anterior–posterior radiographic image was obtained 2 weeks before the administration of ultrasound-guided corticosteroid injection. To ensure the accuracy of the CSA assessment, we adopted a standard imaging protocol involving an upright standing posture and a descending beam tilted at 20°. For the CSA measurement, we adopted the standard protocol reported by Blonna et al. [18] This measurement protocol was used to prevent scapular rotation and projection angle differences. We obtained a true anterior–posterior view without overlapping the edge of the glenoid cavity. The angle of the CSA was measured using the line from the superior and inferior bone margins of the glenoid and the line from the inferior bony margin of the glenoid to the most lateral border of the acromion. To ensure the CSA assessment accuracy, two independent evaluators measured the CSA of the shoulder radiographic images by using the same method, and each measurement was performed thrice. The CSA of each radiographic image was randomly evaluated thrice by these two evaluators. Six CSA image values were obtained for each patient, and the mean value of the six images was recorded for analysis. The inter- and intraobserver reliability for the CSA evaluation were excellent, and the intraclass coefficient was >0.9, as reported by another study [19].

### 2.3. Intervention

Patients were administered a combination of 1 mL of triamcinolone acetonide and 2 mL of lidocaine by using a 23-gauge needle under ultrasound guidance for the treatment of subacromial bursa. The ultrasound evaluation and injection were administered by an experienced clinical physician who was blinded to the CSA of the patients.

### 2.4. Outcome Assessment

Shoulder pain and disability index (SPADI), visual analog scale (VAS), and active ROM scores were obtained as the outcomes of this study. The SPADI is a 13-item self-administered questionnaire used to evaluate the levels of disability and shoulder pain. The total SPADI score ranges from 0 to 100, with higher scores indicating more severe disability and pain [20]. A minimum 8-point difference in SPADI scores was considered significant [21]. The VAS was used to rate the shoulder pain over the week preceding their participation, with the patients scoring their pain from 0 (no pain) to 10 (tremendous pain) [22]. In addition, we assessed active ROM for four directions (forward flexion, abduction, internal rotation, and external rotation) using an electrical goniometer. When the patients reached the end of their active ROM, the goniometer was placed on the distal forearm proximal to the wrist. The patients were asked to slowly move their shoulders until they reached an angle at which the pain became intolerable; the motion was performed thrice, and the median angle value was recorded. These measurements were performed at baseline and at weeks 2, 6, and 12 following the ultrasound-guided corticosteroid injection.

### 2.5. Sample Size

We used G*power (version 3.1.9.2, UCLA, Los Angeles, CA, USA) to perform a preliminary power analysis using the independent *t* test and to identify between-group differences in VAS scores from baseline to 12 weeks after injection. Analyses suggested that a minimum of 52 participants were required to achieve the appropriate power ([1 − β] = 0.80; α = 0.05; because no preliminary data were available, we used a large effect size of 0.80) [23].

### 2.6. Statistical Analysis

Demographic and baseline data were analyzed for each group. Additionally, the radiographic spur size was measured as the distance from the point where the inclination of the anterior edge of the acromion abruptly increased to the tip of the spur. The length of less than 5 mm was defined as small, 5 mm to less than 10 mm as medium, and 10 mm or greater as large according to the classification of Tada et al. [24]. Continuous variables are presented as means with standard deviations; abnormally distributed variables are presented as medians with percentile ranges. Categorical variables are presented as numbers and proportions. The chi-square test was used to analyze categorical demographic data. Continuous variables between groups were compared using an independent Student’s *t* test, and a Kolmogorov–Smirnov test was performed to verify the normality of the distribution. If the data were not normally distributed, we performed the Mann–Whitney U test to compare mean values between the groups at each time point. Repeated-measures analysis of variance with a post hoc Bonferroni test was used for intragroup data at different follow-up time points. A Pearson correlation analysis was performed to identify any correlation between the CSA and clinical outcome parameters at each time point. Analyses were performed using SPSS (version 25.0; IBM, Armonk, NY, USA). All statistical tests were two-tailed, and a *p* value of <0.05 was considered statistically significant.

## 3. Results

**Figure 1:**
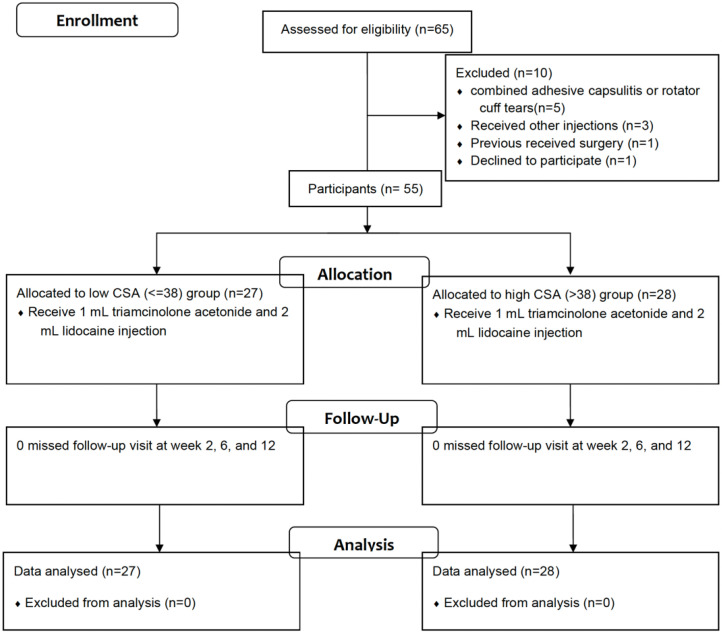
Study flowchart.

The eligibility of 65 patients was evaluated; 10 patients were excluded, and the remaining 55 patients were enrolled in this study (Figure 1).

The patients’ baseline demographic parameters, namely age, sex, body mass index, medication use, physical therapy, and affected side, did not differ between the low-CSA and high-CSA groups (Table 1).

The VAS, SPADI, and shoulder active ROM scores of the two groups throughout the study are presented in Table 2.

At baseline and weeks 2, 6, and 12, VAS, SPADI, and shoulder ROM scores did not differ between the groups. Regarding changes in SPADI and VAS scores, compared with the scores at baseline, both the CSA groups demonstrated significantly decreased scores at weeks 2, 6, and 12; these scores were greater than their minimal clinically important differences scores. Similarly, the two groups’ active ROM improved from baseline after corticosteroid injection. Regarding the correlation between the CSA and clinical parameters at different times, the baseline VAS score demonstrated a positive correlation (*p* = 0.024) with the CSA. However, the VAS score after intervention was not associated with the CSA measured before injection. No correlation was identified at baseline or at weeks 2, 6, and 12 between the SPADI score and CSA. Similarly, the ROM at baseline and at weeks 2, 6, and 12 was not associated with the CSA before intervention (Table 3).

## 4. Discussion

The findings of this prospective case–control study reveal that the CSA was not associated with the efficacy of ultrasound-guided corticosteroid injection in patients with subacromial bursitis. The shoulder VAS, SPADI, and active ROM scores did not differ between the patients with low and high CSAs at 2, 6, and 12 weeks after intervention. When the correlations between the CSAs and all clinical parameters at different times were analyzed, only the baseline VAS score was positively correlated with the CSA. This finding suggests that corticosteroid injection exerted a limited effect on the CSAs of the two groups, and the CSAs were not associated with clinical presentation 12 weeks after intervention.

Our study demonstrated that ultrasound-guided corticosteroid injection was effective for the patients with subacromial bursitis, and the effects of such injections lasted up to 12 weeks. This finding is compatible with that of a previous study, which reported that corticosteroid injection rapidly reduced pain in the first 3 to 12 weeks [25]. The clinical effects of corticosteroid treatment are attributable to its anti-inflammatory characteristics, which can be explained by the pathogenesis of subacromial bursitis, which occurs mainly with inflammation. Other studies have identified the increased expression of substance *p*, vascular endothelial growth factor, and cytokines such as tumor necrosis factor- α, interleukin (IL)-1, and IL-6 in the subacromial bursa of patients with impingement syndrome [26,27,28]. These factors can induce chronic inflammation and increase vascularity in the bursa, causing pain and impingement of the subacromial bursa during shoulder movements. Corticosteroid can suppress inflammation and subsequently relieve the symptoms of subacromial bursitis.

Patients with shoulder pain with a higher CSA had a higher risk of supraspinatus tendinopathy [17]. The CSA can affect the compression and sheer force of the shoulder joint. The sheer force of the joint increases with the CSA, which can lead to instability in the shoulder joint. Subsequently, the higher CSA and shoulder joint instability lead to increased load on the SS tendon of the subacromial space to compensate for shoulder instability [29]. When patients with high CSAs perform abduction, they experience more stress in the subacromial space and impingement of the shoulder. Because of this biomechanical mechanism, repeat subacromial bursa impingement can result in chronic bursitis and lead to pain in the shoulder. Although clinical parameters did not significantly differ between the low- and high-CSA groups in our study, our correlation analysis indicated a positive correlation between the CSA and VAS scores before ultrasound-guided corticosteroid injection. This may be because although the cutoff point of 38° for the CSA was chosen according to the finding of a previous study, it may not be optimal for subgroup analysis. The observation of higher VAS scores in the patients with higher CSAs before injection may be compatible with the theory of subacromial bursa impingement, although no association was identified after injection or in the subgrouping comparison. We propose that the effect of the CSA was not adequately strong to lead to overall different efficacies of corticosteroid injection in patients with subacromial bursitis within 12 weeks of intervention.

Although our study reported negative findings, it is the first to investigate the association between the CSA and the outcomes of ultrasound-guided subacromial injection for subacromial bursitis. Several studies have investigated the effect of radiographic imaging parameters on the outcomes of shoulder injections; however, our study focused on subacromial bursitis and particularly on subacromial bursa corticosteroid injection with ultrasound guidance [30,31]. Our findings are not consistent with those reported by Dietrich et al., who indicated that subacromial injection exhibited higher efficacy in patients with a CSA of >35° than in those with a CSA of <35° at 1 month after injection [30]. Dietrich et al. enrolled patients with several types of shoulder diseases, such as osteoarthritis and adhesive capsulitis, and did not consider other adjunctive therapies, such as physiotherapy and NSAID use. Our study considered the possible clinical effect of physiotherapy and NSAIDs on subacromial bursitis. In addition to pain, we measured the functional aspects of the shoulder after injection, with a 12-week follow-up period. The different outcome in our study could be due to our study participants and design, which differed from those of Dietrich et al.

The main limitation of this study is the limited follow-up period. Although the follow-up period after intervention was 3 months, a longer period may reveal that different CSAs in patients with subacromial bursitis have different levels of effects. An additional limitation is that MRI examination, which can be used to identify labral lesions and identify patients with comorbidities for exclusion, was not performed due to limited research funds. However, in consideration of this limitation, we performed ultrasound and physical examinations and reviewed patients’ shoulder trauma histories and exercise habits. No participants had histories of shoulder trauma or sports-related lesions. We assume that undetected labral lesions would be rare among these participants. Finally, the CSA cutoff for subgrouping was 38°, which was determined according to a previous study [29]. Because no study has reported on the association between CSAs and subacromial bursitis, we conducted a study to investigate the accuracy of predicting supraspinatus tendinopathy based on CSAs. Differences among different races may lead to different optimal cutoff CSAs for grouping and therefore different outcomes. To account for the potential CSA grouping bias, we performed additional analyses of the correlation between CSAs and clinical parameters. The CSA was not correlated with clinical presentation after ultrasound-guided subacromial corticosteroid injection among patients with subacromial bursitis.

## 5. Conclusions

The CSA was not associated with the clinical efficacy of ultrasound-guided subacromial corticosteroid injection in patients with subacromial bursitis after a 3-month follow-up period. Other possible contributing factors regarding the prognosis after ultrasound-guided corticosteroid injection should be considered in patients with subacromial bursitis. In addition, studies with longer follow-up periods should be conducted to verify the effect of the CSA.

## Figures and Tables

**Table 1:** Demographic and clinical characteristics at baseline.

Variables	All Participants, *n* = 55	CSA ≤ 38°, *n* = 27	CSA > 38°, *n* = 28	*p* Value
Age (years)	56.1 (11.4)	57.4 (12.4)	54.8 (10.4)	0.399
CSA (degrees)	38.2 (5.4)	33.7 (3.4)	42.5 (2.9)	<0.001
Sex (female/male)	31/24	12/15	19/9	0.106
BMI	24.1 (2.9)	24.6 (2.8)	25.4 (3.3)	0.305
Medication for pain (yes/no)	25/30	12/15	13/15	0.788
Physical therapy (yes/no)	30/25	15/12	15/13	0.788
Affected (left/right)	21/34	10/17	11/17	1.000
Spur (none/small/medium/large)	30/20/5	16/9/2	14/11/3	0.773

CSA, critical shoulder angle. *p* value according to independent Student’s *t* test or Mann–Whitney U test.

**Table 2:** Outcome measurements at baseline 2, 6, and 12 weeks after intervention.

Measurements	CSA ≤ 38°, *n* = 27, Mean ± SD	Mean Difference (95% CI)	*p* Value ^a^	CSA > 38°, *n* = 28, Mean ± SD	Mean Difference (95% CI)	*p* Value ^a^	*p* Value ^b^
VAS baseline	6.3 (0.9)			6.6 (1.1)			0.301
Week 2	5.2 (1.0)	−1.1 (−1.4 to −0.7)	<0.001	4.7 (1.4)	−1.9 (−2.6 to −1.2)	<0.001	0.127
Week 6	3.4 (1.3)	−2.9 (−3.7 to −2.1)	<0.001	3.4 (1.2)	−3.2 (−4.0 to −2.3)	<0.001	0.966
Week 12	3.9 (1.1)	−2.3 (−3.1 to −1.6)	<0.001	3.5 (1.0)	−3.1 (−3.9 to −2.3)	<0.001	0.153
SPADI baseline	60.2 (10.1)			62.0 (10.3)			0.529
Week 2	47.7 (9.7)	−12.6 (−15.2 to −9.9)	<0.001	46.4 (12.8)	−15.6 (−20.9 to −10.3)	<0.001	0.680
Week 6	39.7 (10.2)	−20.6 (−25.3 to −15.8)	<0.001	36.5 (10.2)	−25.4 (−31.5 to −19.4)	<0.001	0.261
Week 12	37.0 (11.0)	−23.2 (−28.5 to −18.0)	<0.001	33.4 (8.5)	−28.6 (−34.5 to −22.7)	<0.001	0.179
Flexion baseline	137.8 (15.6)			131.5 (25.2)			0.513
Week 2	162.0 (8.0)	24.3 (13.8 to 34.8)	<0.001	162.7 (9.3)	31.2 (17.4 to 45.3)	<0.001	0.764
Week 6	163.3 (7.1)	25.6 (16.6 to 34.5)	<0.001	162.9 (6.6)	31.3 (16.3 to 46.3)	<0.001	0.797
Week 12	147.8 (7.7)	10.0 (0.4 to 19.7)	0.038	150.2 (8.9)	18.7 (4.7 to 32.6)	0.004	0.297
Abduction baseline	133.9 (19.5)			130.1 (18.2)			0.512
Week 2	163.6 (9.0)	29.6 (17.8 to 41.7)	<0.001	162.8 (9.3)	32.2 (20.8 to 43.6)	<0.001	0.744
Week 6	163.9 (7.6)	30.0 (18.2 to 40.6)	<0.001	161.6 (7.0)	31.0 (20.5 to 41.6)	<0.001	0.251
Week 12	145.1 (11.3)	11.2 (−0.7 to 23.1)	0.075	146.3 (14.4)	15.8 (3.3 to 28.3)	0.008	0.729
Internal rotation baseline	44.1 (9.7)			46.4 (12.8)			0.956
Week 2	56.3 (6.4)	12.2 (7.0 to 17.4)	<0.001	56.4 (4.9)	12.5 (6.6 to 18.4)	<0.001	0.932
Week 6	57.1 (5.0)	14.1 (8.9 to 6.5)	<0.001	58.2 (5.5)	14.3 (8.0 to 20.6)	<0.001	0.963
Week 12	54.4 (4.4)	10.4 (4.9 to 15.8)	<0.001	53.4 (5.0)	9.5 (4.5 to 14.4)	<0.001	0.410
External rotation baseline	54.4 (9.7)			56.1 (10.3)			0.550
Week 2	63.0 (4.9)	8.5 (3.8 to 13.2)	<0.001	63.4 (5.8)	7.3 (1.9 to 12.7)	0.004	0.767
Week 6	63.9 (5.3)	9.4 (3.4 to 15.5)	0.001	62.9 (5.4)	6.8 (0 to 13.6)	0.051	0.474
Week 12	60.7 (4.7)	2.1 (0.4 to 12.2)	0.033	61.6 (4.5)	5.5 (−0.6 to 11.7)	0.101	0.491

^a^ Intragroup analysis (analysis of variance for repeated measurements); ^b^ Intergroup analysis (Student’s *t* test).

**Table 3:** Pearson correlation of CSA and clinical parameters at baseline and 2, 6, and 12 weeks after intervention.

Measurements	Baseline	Week 2	Week 6	Week 12
VAS	0.304 *	−0.067	0.007	−0.147
SPADI	0.048	−0.112	−0.248	−0.220
Flexion	−0.081	0.039	0.069	−0.072
Abduction	−0.107	0.120	−0.116	−0.026
Internal rotation	−0.150	−0.105	−0.036	−0.073
External rotation	−0.045	0.050	0.070	0.131

* Significant correlation of critical shoulder angle, *p* < 0.05.

## Data Availability

The datasets used and/or analyzed during the current study are available from the corresponding author on reasonable request.
